# A200 A POPULATION-BASED ASSESSMENT OF PHYSICAL ACTIVITY AND EXERCISE IN PERSONS WITH IBD

**DOI:** 10.1093/jcag/gwae059.200

**Published:** 2025-02-10

**Authors:** C L Dolovich, S Chochinov, G Ly, A Oketola, S Narvey, S Larance, M Raman, S Webber, C N Bernstein

**Affiliations:** Internal Medicine, University of Manitoba Max Rady College of Medicine, Winnipeg, MB, Canada; Internal Medicine, University of Manitoba Max Rady College of Medicine, Winnipeg, MB, Canada; University of Manitoba, Winnipeg, MB, Canada; University of Manitoba, Winnipeg, MB, Canada; Internal Medicine, University of Manitoba Max Rady College of Medicine, Winnipeg, MB, Canada; University of Manitoba, Winnipeg, MB, Canada; University of Calgary, Calgary, AB, Canada; University of Manitoba College of Rehabilitation Sciences, Winnipeg, MB, Canada; University of Manitoba Max Rady College of Medicine, Winnipeg, MB, Canada

## Abstract

**Background:**

It is known that physical activity (PA)/exercise is associated with a reduced risk in chronic disease. However, less is known about its impact on inflammatory bowel disease (IBD) specifically. Research generally uses the terms ‘PA’ and ‘exercise’ interchangeably, which although similar, are overlapping classifications of activity. As a result, interpreting and comparing existing findings can be challenging.

**Aims:**

We aimed to assess the physical activity and exercise profiles of persons with IBD, their limitations in undertaking PA/exercise, and how PA correlates with active disease and fatigue in a population-based sample in Manitoba, Canada.

**Methods:**

A cross-sectional survey study was undertaken by participants in the University of Manitoba IBD Research Registry. The survey included sociodemographic factors, PA/exercise, the International Physical Activity Questionnaire (IPAQ), Inflammatory Bowel Disease Symptom Inventory (IBDSI), and Modified Fatigue Impact Scale (MFIS). Bivariate and logistic regression analyses were used to assess the association between PA and disease activity and fatigue.

**Results:**

The survey was completed by 1257 of 2740 invitees (45.8%). The mean age of participants was 60.8 +/- 13.0 years. The most common type of PA and exercise that respondents participated in were walking for leisure/exercise (84%) and the most common type of PA and exercise avoided was running/jogging (16%). Physically active status per IPAQ was categorized as inactive-low in 42.5%, moderately active in 32.0%, and highly active in 25.5%. Average fatigue levels were mean 24.9 SD 18.9 per MFIS. PA and fatigue levels were similar for those >55 vs those <55 years. For ulcerative colitis (UC) persons, after adjusting for demographic and clinical factors, the odds of lower levels of PA were greater among females [aOR 95% CI, 1.76 (1.12-2.78)], ever smoked [aOR 95% CI, 2.55 (1.11-5.73)], active disease (per IBDS) [aOR 95% CI, 1.68 (1.07-2.62)], and with an increase in MFIS total score [aOR 95% CI, 1.02 (1.01-1.04)].

**Conclusions:**

There was a high rate of inactive to low PA among persons with IBD. Females with UC especially need to be encouraged to increase their PA. While it is not surprising that persons with active disease may have reduced or even avoided PA, this requires further study. Since as a result, PA may reduce deconditioning that may accompany periods of active disease and increased fatigue. These findings underscore the need for tailored PA/exercise guidelines for individuals with IBD. Future research should explore objective measures of PA to better understand PA/exercise in both younger and older adult populations.

**Funding Agencies:**

CAG, None

